# Evaluation of surgical procedures of mouse urethra by visualization and the formation of fistula

**DOI:** 10.1038/s41598-020-75184-5

**Published:** 2020-10-26

**Authors:** Taiju Hyuga, Daiki Hashimoto, Daisuke Matsumaru, Shinji Kumegawa, Shinichi Asamura, Kentaro Suzuki, Kei-ichi Katayama, Shigeru Nakamura, Hideo Nakai, Gen Yamada

**Affiliations:** 1 Department of Developmental Genetics, Institute of Advanced Medicine, Wakayama Medical University, Kimiidera 811-1, Wakayama City, Wakayama 641-8509 Japan; 2 Laboratory of Hygienic Chemistry and Molecular Toxicology, Gifu Pharmaceutical University, 1-25-4 Daigaku-nishi, Gifu-City, Gifu 501-1196 Japan; 3 Department of Plastic and Reconstructive Surgery, Wakayama Medical University, Kimiidera 811-1, Wakayama City, Wakayama 641-8509 Japan; 4 Department of Molecular Genetics, Wakayama Medical University, Kimiidera 811-1, Wakayama City, Wakayama 641-8509 Japan; 5 Department of Pediatric Urology, Children’s Medical Center Tochigi, Jichi Medical University, 3311-1 Yakushiji, Shimotsuke-City, Tochigi 329-0498 Japan

**Keywords:** Anatomy, Urology

## Abstract

Visualization of the surgically operated tissues is vital to improve surgical model animals including mouse. Urological surgeries for urethra include series of fine manipulations to treat the increasing number of birth defects such as hypospadias. Hence visualization of the urethral status is vital. Inappropriate urethral surgical procedure often leads to the incomplete wound healing and subsequent formation of urethro-cutaneous fistula or urethral stricture. Application of indocyanine green mediated visualization of the urethra was first performed in the current study. Indocyanine green revealed the bladder but not the urethral status in mouse. Antegrade injection of contrast agent into the bladder enabled to detect the urethral status in vivo. The visualization of the leakage of contrast agent from the operated region was shown as the state of urethral fistula in the current hypospadias mouse model and urethral stricture was also revealed. A second trial for contrast agent was performed after the initial operation and a tendency of accelerated urethral stricture was observed. Thus, assessment of post-surgical conditions of urogenital tissues can be improved by the current analyses on the urethral status.

## Introduction

Improvement of surgical procedures for the mouse anterior/posterior urethra is vital for urology and modern medical sciences. It is required not only to develop urethral surgical procedures but also to offer information about the post-surgical status of urogenital tissues in vivo*.*

Hypospadias is one of the major human birth defects with an increasing frequency. In the Western countries, its frequency in the entire birth defects has been often reported as approaching to 0.5% of the total population depending on the region^1,2^. Currently only hypospadias repair surgery, urethroplasty, has been adapted to treat it^3^. Such surgery is required to induce minimum post-surgical complications. Urologists have improved various hypospadias surgical procedures including modification of suture performance and formation of neo-urethra^4–6^. Efficient tissue wound healing including the epithelia and surrounding mesenchyme is necessary after such procedures. Even after performing improved surgical procedures, urethral abnormalities such as urethral fistula/stricture have been reported to occur^6–9^. Hence, evaluation of the urethral status after post-urethral surgery is essential to improve surgical outcomes.

Various surgical animal models have been reported in urogenital organ^10,11^. In order to evaluate the efficacy of surgical procedures and post-surgical complications, visualization of the operated urethra, urinary tract in post-surgical conditions are essential^12^. Up to now, histological analyses including Hematoxylin–Eosin stain have been adapted to analyze urethral epithelia and surrounding connective tissues. Histological analyses can only observe the fixed tissues but not in physiological conditions. Hence, procedures for visualization processes are vital to improve the surgical procedure efficacy in the living animal models.

Here, we report visualization procedures for the status of mouse urethra by indocyanine green (ICG) or X-ray contrast agent (CA). Successful detection of the anterior/posterior urethra and aberrant formation of fistula is demonstrated by CA-mediated visualization. Urethral stricture was also revealed by the current visualization. A second trial for CA-mediated urethral visualization was performed after the initial operation and subsequent awakening. A tendency of accelerated urethral symptom, stricture, was observed which suggests the efficacy of current procedures to defect the time-course of post-surgery status. CA-mediated visualization can give us a clear evaluation of urethral surgical procedures and useful for its development.

## Results

### ICG-mediated visualization of mouse bladder and urethra

**Figure 1:**
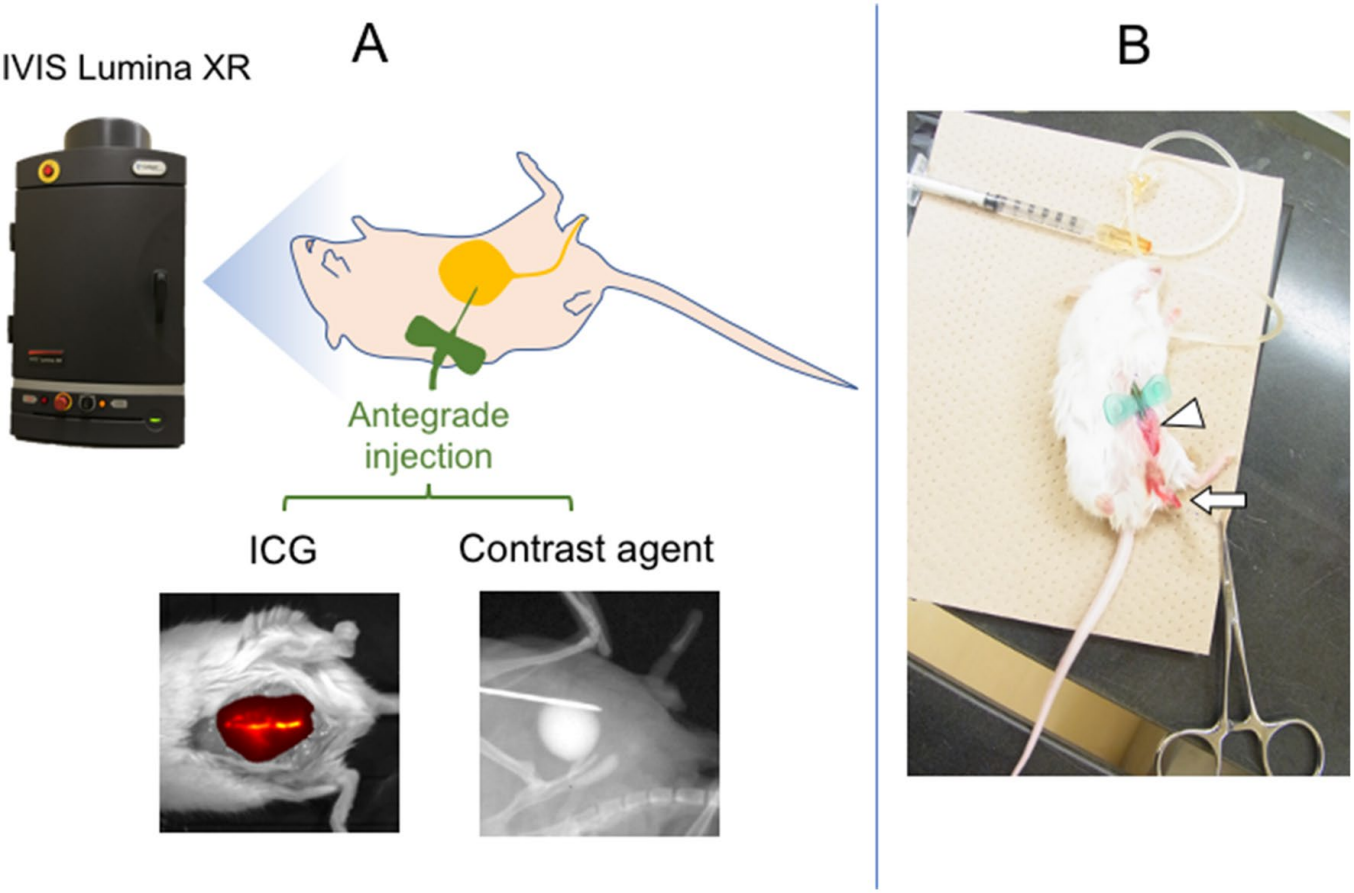
An illustration showing the experimental diagram. (**A**) Surgical procedures and subsequent analyses on the status of operated bladder and ureter/urethra are shown. Various experimental strategies including ICG and CA-mediated visualization were adapted to analyze the status of bladder and urethra. After performing anesthesia and surgical procedures, in vivo imaging utilizing IVIS imaging devise was performed with various parameters of exposure time. Antegrade urethrography was performed. (**B**) After the general anesthesia and surgical procedures (white arrowhead), the mouse was placed oblique position and penis was fixed with tension by 7-0 PDS stay suture (white arrow) and CA was injected through butterfly needle.

**Figure 2:**
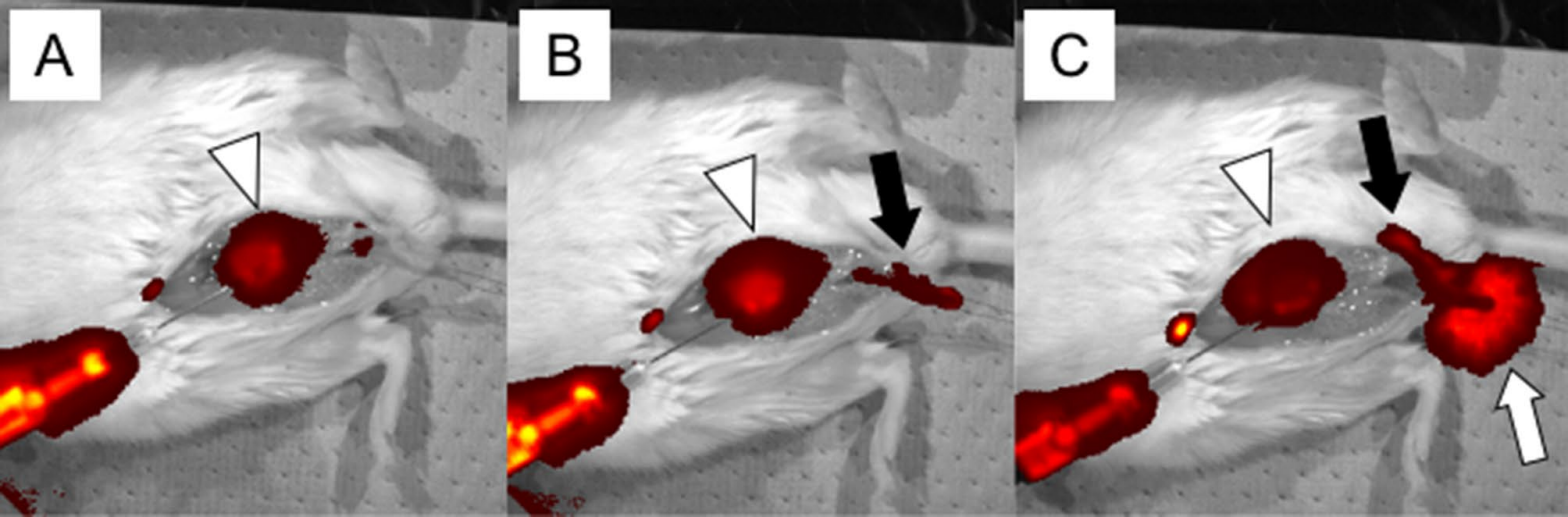
Demonstration of the status of ICG retention in the bladder and urethra. ICG was injected into the bladder. The retained ICG solution was subsequently figured by utilizing in vivo imaging device IVIS. Subsequent amount of retained ICG can be visualized in the bladder and urethra (anterior and posterior). Immediately after the injection, the entire bladder (white arrowhead) was visualized. (**A**) Later, the urethra (black arrow) was visualized in addition to the bladder. (**B**) Urine including ICG overflowed from external urethral meatus (**C**; white arrow).

Injection of ICG has been utilized to visualize the status of tubular structure such as the status of lymphatic vessels after their surgeries^13–15^. Hence, application of ICG-mediated visualization of the urethra was first performed in the current study (Fig. 1A). ICG-mediated visualization revealed the entire bladder image but not the urethral status in mouse pelvis (Fig. 2A–C). Faint signals were detected around the penis (glans) region containing urethra, but ICG signal was not detected in the posterior urethra (Fig. 2B,C).

### CA-mediated visualization of mouse bladder and urethra

**Figure 3:**
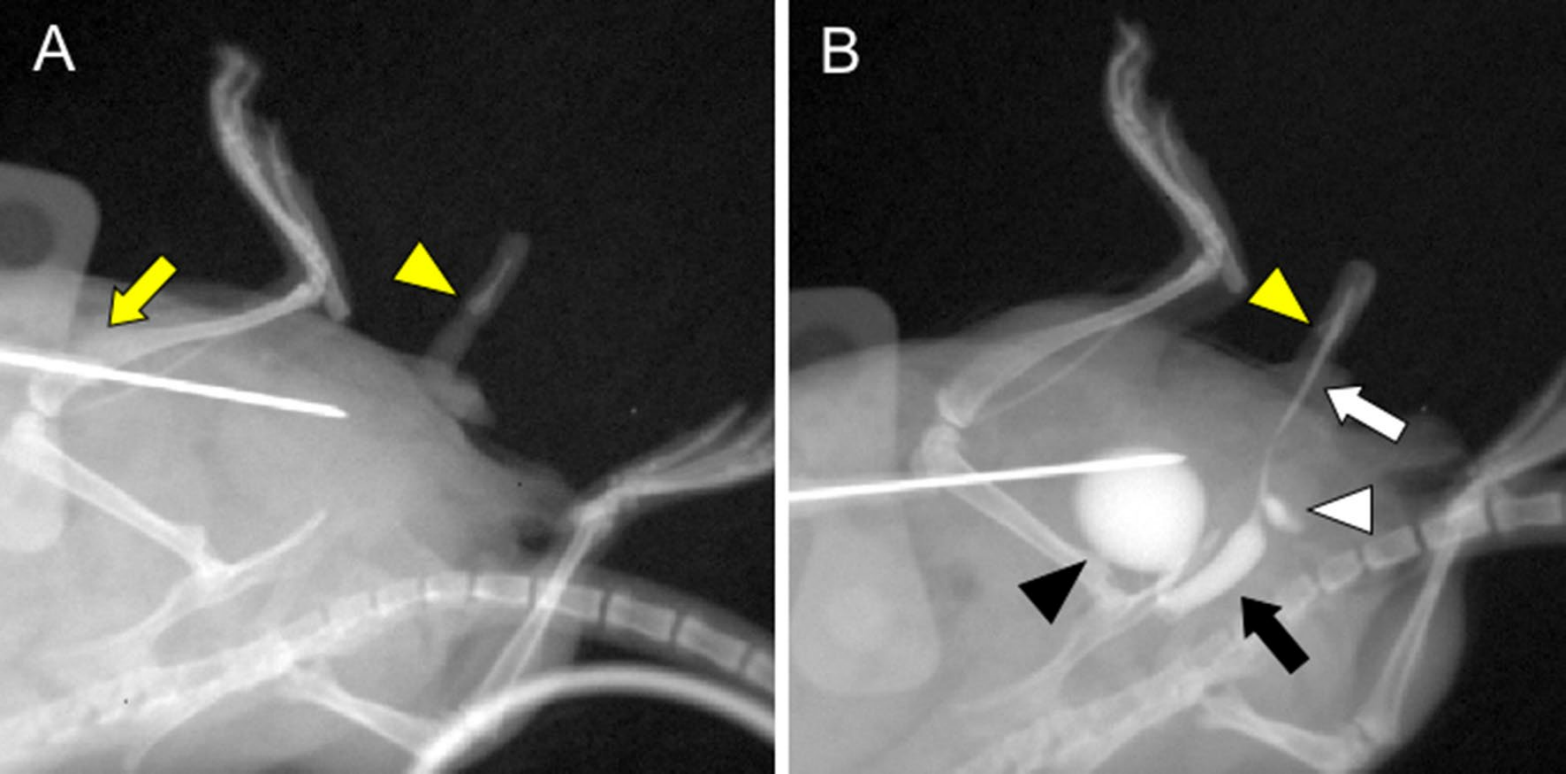
The urinary tract status including bladder and urethra in vivo was detected by the current procedure of anterograde CA-mediated urethrography. This examination was performed in oblique position with normal laboratory mouse, similar to urethrography for human. Pre-examination image (**A**) with butterfly needle (yellow arrow) and os penis (yellow arrowhead) before CA injection is shown. Visualization was performed by the CA injection to the bladder through butterfly needle. (**B**) Bladder (black arrowhead), anterior urethra (white arrow), posterior urethra (black arrow) and urethral diverticulum (white arrowhead) were visualized. The caliber of the posterior urethra was wider than that of the anterior urethra (black and white arrow) and the border between them was distinguished at the urethral diverticulum (white arrowhead). The position of the os penis (yellow arrowhead) was the landmark in distinguishing between the part of penile shaft and grans.

In order to access and visualize the urethral status, CA was injected and X-ray mediated urethral analysis was performed utilizing IVIS Imaging System (Fig. 1A,B). Antegrade injection of CA into the bladder enabled to detect urethral status in vivo (Fig. 3 A, B). Retrograde mediated injection trial from the urethral meatus failed to detect such structure (data not shown). The caliber of the posterior urethra was clearly wider than that of the anterior urethra (Fig. 3B; black arrow and white arrow). The border between posterior and anterior urethra was shown around the urethral diverticulum. The position of the os penis (Fig. 3B; yellow arrowhead) was the landmark to distinguish penile urethra from granular urethra and the glandular urethra was visualized in distal side of os penis^16^.

### Surgical models for hypospadias-like incision to the mouse urethra

**Figure 4:**
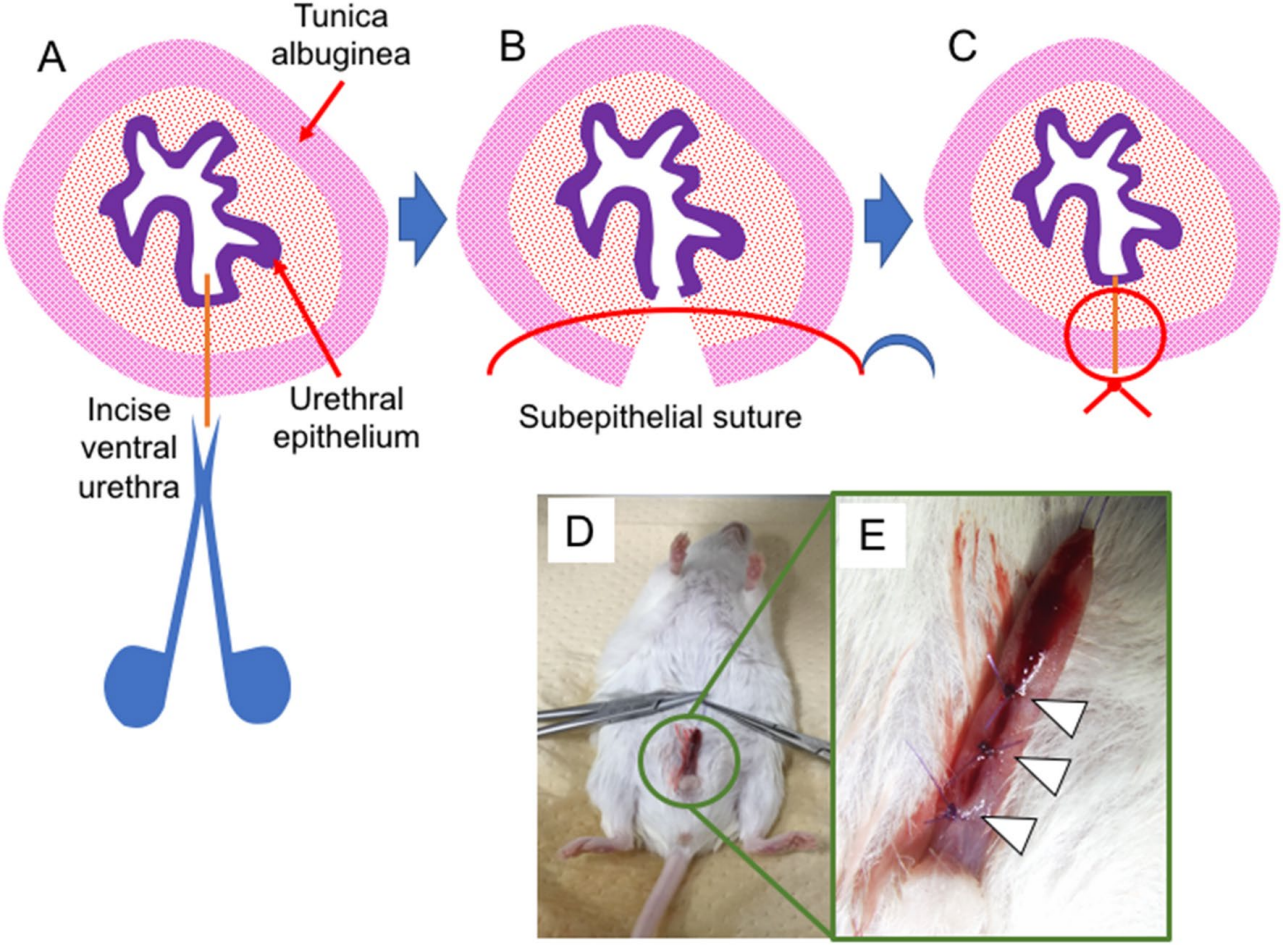
The procedure of the hypospadias-like surgery is demonstrated. After the general anesthesia, ventral urethra was vertically incised by ophthalmic scissor. (**A**) Urethroplasty was performed with sub-epithelial interrupted suture. (**B**,**C**) Post-operative surgical sites (3 interrupted sutures; white arrowheads) are shown (**D**,**E**).

Hypospadias-like model was reported in several animal models^17–19^. Many of them adopted urethral full-thickness incision. Thus, 3 mm vertical skin incision was placed in the dorsal prepuce of penis. The glans was exposed externally by this procedure and an absorbable suture (7-0 PDS) was placed into it for traction. Using ophthalmic scissor, the ventral urethra was incised with full-thickness (approximately 10 mm in length) from the meatus up to the base of penis vertically (Fig. 4A). The external urethral meatus was placed proximally. Sub-epithelial suture was utilized in urethral closure (Fig. 4B,C). The urethroplasty was performed for tubularization by three interrupted one-layer suture (7–0 PDS) at approximately 1.5 mm interval (Fig. 4D,E; white arrowhead).

### The post-surgical urethral status in the hypospadias-like model visualized by the CA-mediated imaging

**Figure 5:**
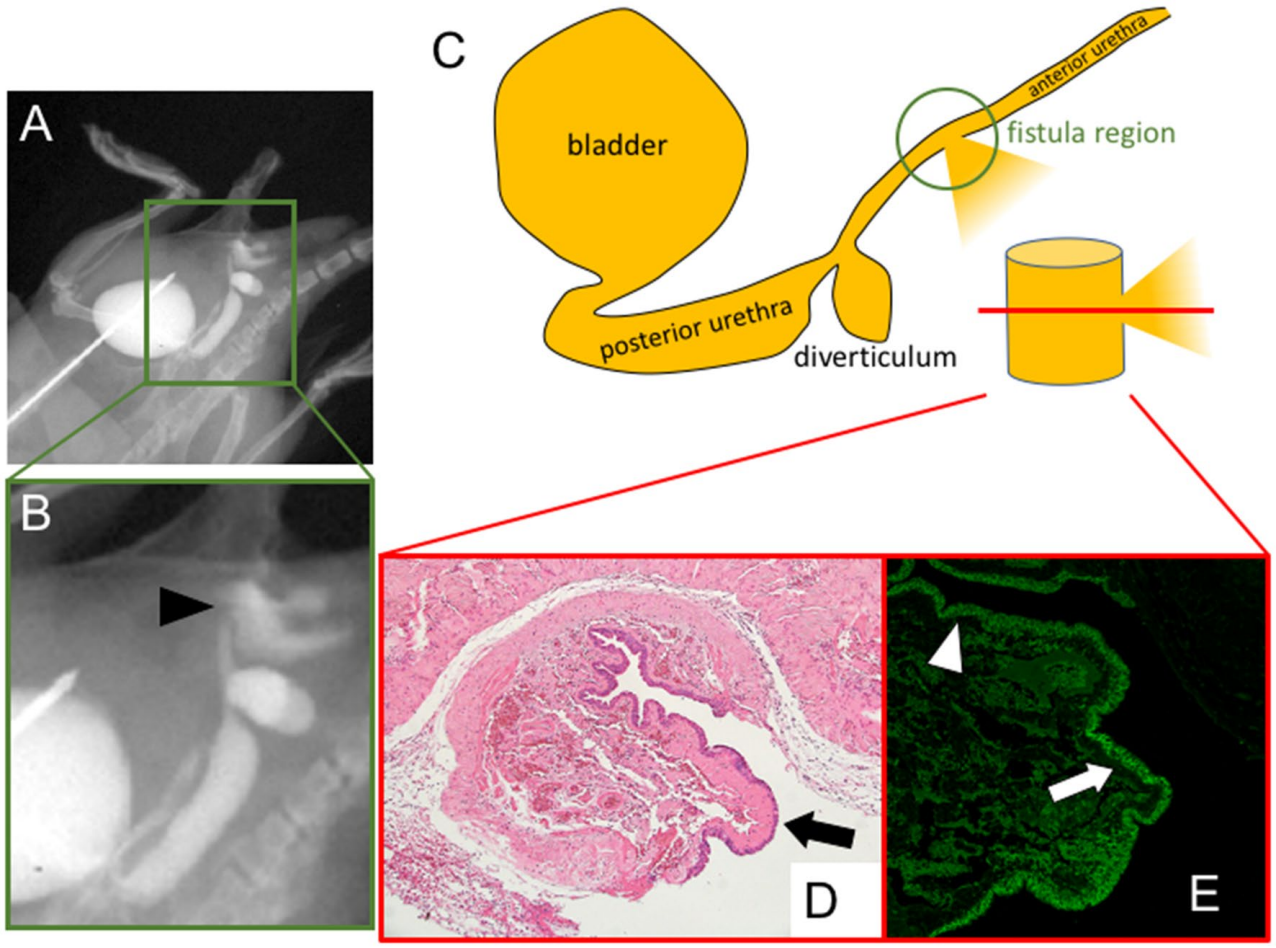
Detection of anterior and posterior urethral status in vivo by the current procedure of anterograde CA injection to the bladder by IVIS imaging. The formation of fistula (black arrowhead) after 2 days of operation are shown (**A**, **B**). Histological examination of epithelial status of the operated site (**C**; green circle) in axial section (C; red line section). Hematoxylin Eosin (**D**; black arrow) and anti-E-Cadherin (**E**; white arrow) stain revealed the epithelialization of the elapsed fistula region (**D**,**E**). E-Cadherin was also expressed in the native urethra (white arrowhead).

Visualization of the urethra and leakage of CA from the operated region was shown after 2 days of operation. The elapsed region was shown as the state of urethral fistula (Fig. 5A,B; black arrowhead in B). Presence of the epithelialization in the elapsed fistula region was revealed by histological analysis (Fig. 5C–E). Hematoxylin Eosin stain revealed the epithelialization of the elapsed fistula region (Fig. 5D; black arrow). E-Cadherin was expressed in the native urethra (Fig. 5E; white arrowhead) and the elapsed fistula region (Fig. 5E; white arrow).

### Surgical models of the mouse urethral stricture demonstrate the morphology of anterior and posterior urethra by CA-mediated visualization

**Figure 6:**
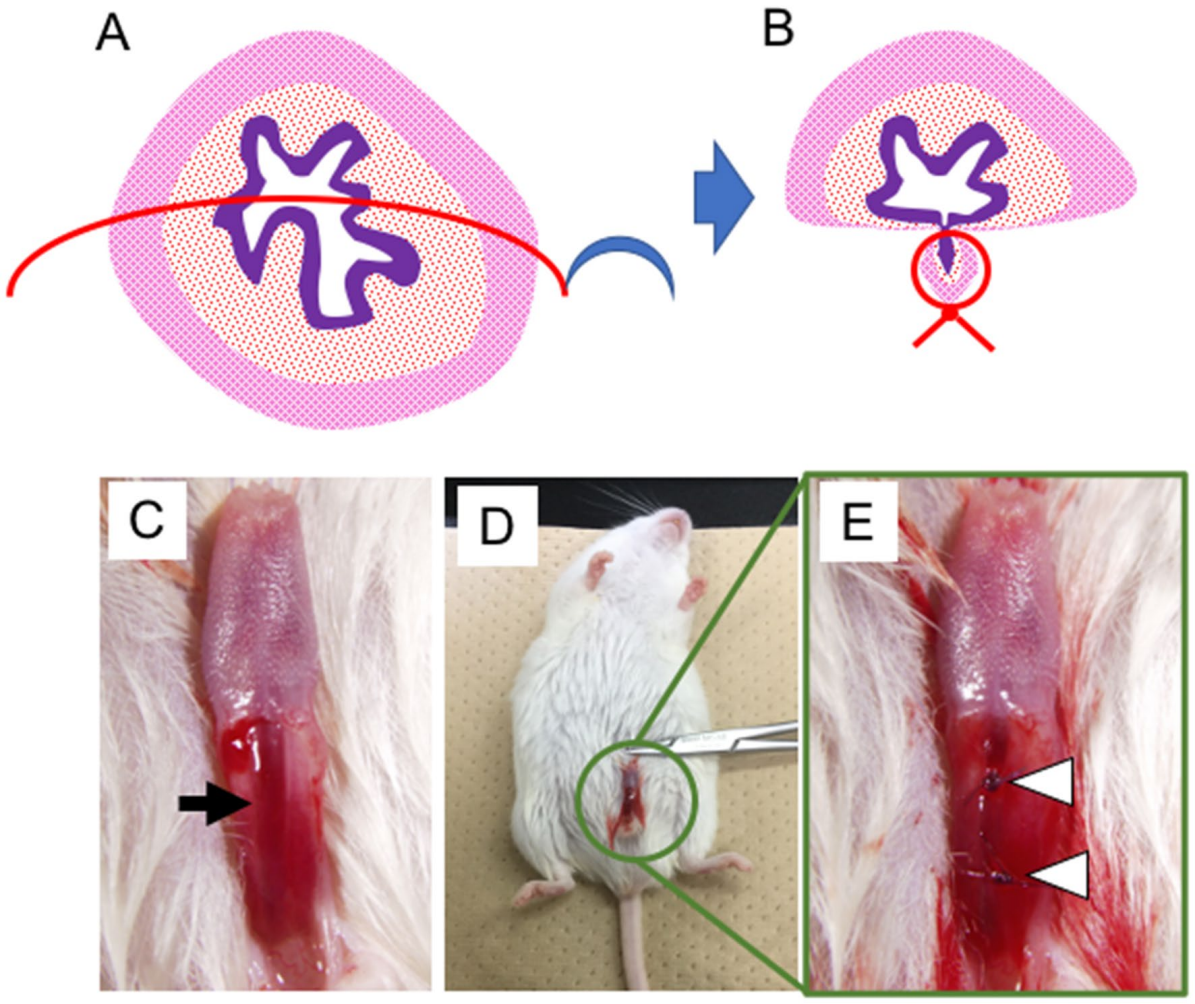
The procedures of the surgical urethral stricture model are shown (**A**,**B**). Prepuce was removed and the urethra was exposed (**C**; black arrow). The blind 7–0 PDS suture was performed at two points of urethra (white arrowhead). Post-operative sites are shown (**D**,**E**).

**Figure 7:**
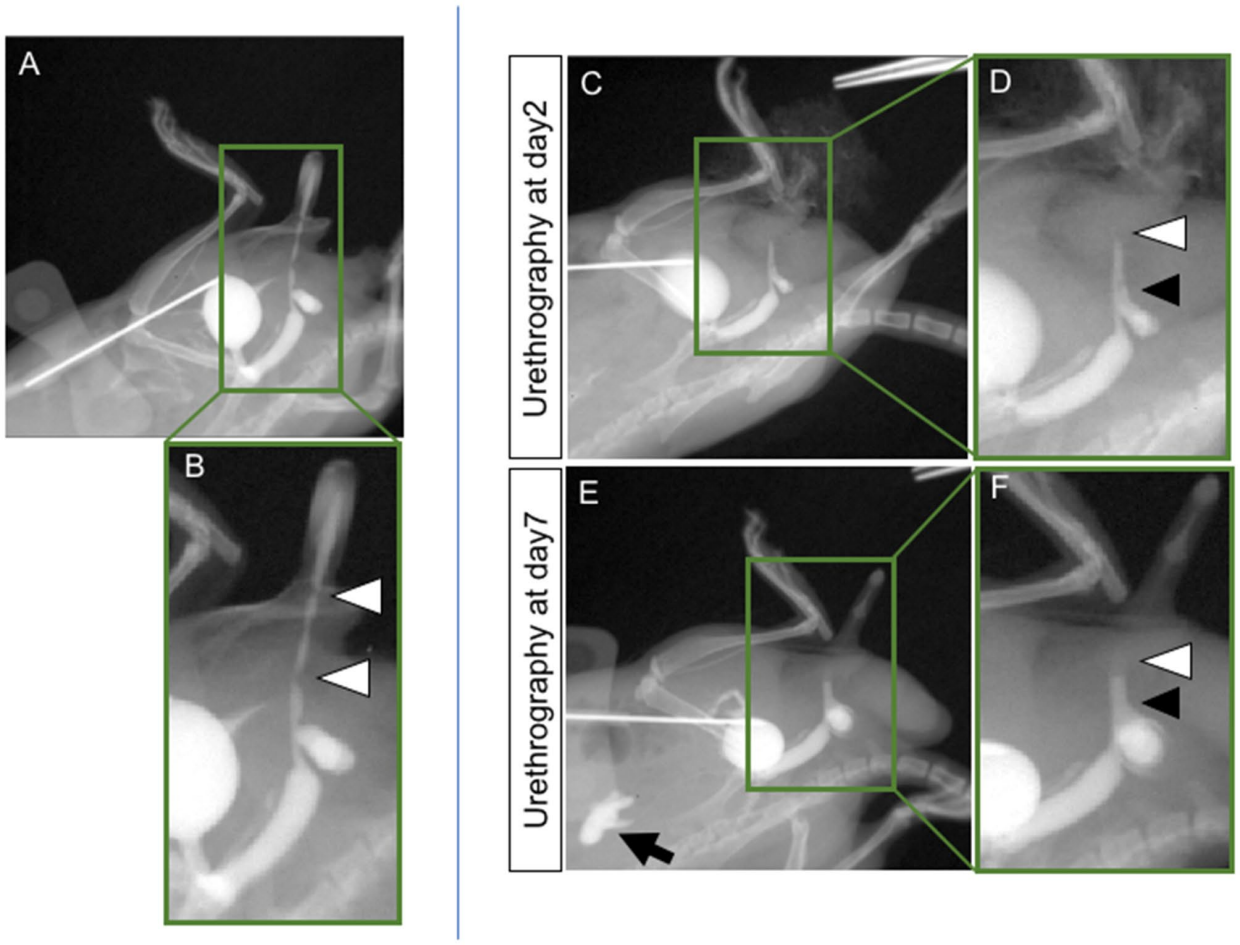
The antegrade urethrography images of urethral stricture model after 2 days of operation (**A**,**B**). Two urethral regions without CA signals are shown (white arrowheads). The strictures of these regions are affected by urethral sutures. First (post-operative 2 days) and second (post-operative 7 days) round urethrography (**C**–**F**). Urethral strictures are shown by white arrowheads. Subsequently, the proximal side of urethra was prominently dilated (black arrowhead). Vesicoureteral reflux was revealed clearly in urethrography (post-operative 7 days; black arrow).

The current model was established to identify the urethral stricture by narrowing the urethral caliber (Fig. 6A,B). 3 mm vertical skin incision was placed in the dorsal prepuce of penis and the glans was exposed externally. A 7-0 PDS suture was placed into the glans for traction. The prepuce over the urethra was removed and urethra was exposed (Fig. 6C). The blind 7-0 PDS suture for urethra was performed at the two points so that suture was passed through 3 and 9 o’clock position (Fig. 6A,B,D,E; white arrowheads). The anterior urethra, which shows the urethral stricture as post-hypospadias complication, was selected as the surgical site. The surgical procedure was relatively easier to perform in anterior urethra than posterior urethra. Urethral stricture was also revealed by the current visualization after 2 days of operation. Two urethral regions without CA signals are shown (Fig. 7A,B; white arrowheads). These regions were affected by urethral sutures. Urethrography was performed by the same procedure 5 days later. A second trial for CA induced urethral visualization was performed after the initial operation and subsequent awakening. A tendency of accelerated urethral symptom, stricture, was observed after repeated visualization (Fig. 7C–F). Enlarged urethral caliber was noted (black arrowhead) adjacent to the stricture (white arrowhead).

## Discussion

Assessment of post-surgical conditions of defective pediatric tissues such as urethra can be improved by the analyses on the status of tissues. Visualization of in vivo status of the urethra offers vital information to evaluate the efficacy of surgical procedures. The status of the urethra depends on physiological parameters. Mouse urinates 10–15 times per day physiologically (with each urination containing 0.2 ml)^20^. Inappropriate urethral surgical procedure for infants often leads to the incomplete wound healing resulting in urethral inflammation and subsequent formation of urethro-cutaneous fistula or urethral stricture.

Visualization of the status of anterior and posterior urethra in vivo is achieved by utilizing imaging device. Applications of ICG-mediated visualization has been often utilized in clinics for the operated lymphatic vessels^21^. For first series of trials, application of ICG procedures is examined in mice. Only the presence of the organ such as bladder was detected by ICG-mediated visualization. It has been reported that ICG-mediated visualization detected rather broad signals around the injected site^14^. Similarly, signals around the penis were also detected but not in the posterior urethra. ICG-mediated visualization for bladder or other tissues was reported in large experimental animals such as the case of rat^14^. However, no studies have been reported for small animal models such as the case of mice, particularly for urethra. The evaluation of urethra in ICG has not been reported even in large animals. Regarding the treatment of urethral stricture, it will be useful for detecting the region of urethral stricture in large animals by real-time ICG imaging.

Human urethra is divided as prostatic (posterior), membranous, spongy (anterior) parts^22^. About mouse urethral definition, detailed studies were not reported. Experimental study of urethrography has not been performed in mouse models to our knowledge. Detection of anterior and posterior urethral status in vivo becomes successful by the current procedure of anterograde CA injection to the bladder and visualization by IVIS LUMINA XR imaging device. The current study is not directly an anatomical study for urethra indicating that posterior and anterior part of mouse urethra are divided by urethral diverticulum. Retrograde injection mediated visualization for presence of urethra has been reported mainly in larger animal models^23^. Retrograde injection fails to detect the proper visualization of the distal region of urethra because mice possess relatively small external urethral meatus (data not shown).

The current CA-mediated analysis possesses several advantages for diagnosis. It has been generally described that anterograde CA injection into the bladder visualizes the state of urethra under the physiological conditions because urination induces dynamic change of its status including its diameter enlargement.

It can be also noted that the current method also possesses limitation. The observation can be only performed under the condition of anesthesia. Mice are relatively small experimental animal models often with defined tissue abnormality such as models for urethra and genitalia. Surgical procedure for the urethra has been often developed in larger experimental animals such as the cases of porcine, rabbits^17–19^. “Second round” of CA injection and subsequent urethral visualization were also performed and there was a tendency of acceleration of urethral symptom, stricture and associated urethral dilation, were observed. This sort of experiment could be useful as analyzing the time-course of post-surgery status. Possible future applications will be observing urethral status in vivo with protocols for some surgery procedures in model animals.

Various imaging methods have been used in urological research fields^24^. Among them, cystourethrography using CA is often performed in the human clinics and is useful for the evaluation of lower urinary tract dysfunction, urethral stricture or urethral injury^25,26^. In children, it is often used voiding cystourethrography for detection of posterior urethral valves^27–29^.

Fistula formation is one of the critical pathogenic status of urogenital tissue after surgical procedures, urethroplasty, such as the case of hypospadias surgery. In human hypospadias repair, full-thickness suture is not utilized and sub-epithelial suture is instead accepted. In pediatric surgical procedures, fistula has been often examined by visual inspection. Depending on different degree of hypospadias, ectopic urethral meatus, and subsequent formation of fistula, has been noted. Fistula has been often described between abdominal skin as the urethro-cutaneous fistula. Formation of fistula may be caused by the rupture leading to the leakage of CA. Hence, “simple” leakage of CA may be considered as a rupture due to incomplete suture of the operated site and the epithelialization of the fistula may occur subsequently. Therefore, observation and definition between fistula and CA leakage should be improved further in the field of surgery. The current procedure detected rather clear shape of CA images confirming the presence of the epithelialization. This suggest that the current visualization may help to examine the fistula in contrast to the CA leakage at the operated site.

Other urethral abnormality such as urethral stricture can be also visualized by the current procedures. Urethral fistula and stricture are suggested to be caused by some urethral surgeries^30^. Urethral stricture and protrusion of the operated site may often lead to the formation of fistula. Hence, possible transitions among such conditions may also be monitored by the current procedures.

Urethral suture mediated recovery is designed to prevent the leakage of CA from the operated site. It can be possible that injected CA leakage tended to occur in the intermediate region between the step-wised sutures sites. Extensive sutures such as running suture may prevent the formation of such CA leakage at the operated site. Examination of such tendency of leakage related with step-wise and more intensive treatments including running suture would be necessary. Further examination on such parameters will contribute to improve surgical procedures by animal models.

## Conclusions

1. ICG-mediated visualization of mouse penis and bladder is performed. The procedure is not adequate to identify the urethral status and its diameter.
2. CA-mediated visualization revealed the shape of urethra and the formation of urethral fistula.
3. The formation of fistula was observed by modulating surgical procedures such as suture intervals along the urethra. Such examinations are useful to analyze various fistula formations for post-surgical procedures.
4. Urethral abnormalities such as urethral stricture is also revealed and a second trial for CA induced urethral visualization is performed after the initial operation and subsequent awakening. A tendency of accelerated urethral symptom, constriction, is observed.
5. Improvement of surgical procedures by CA-mediated visualization will contribute for the development of surgical procedures.

## Methods

### Mice

Mice are purchased from Japan SLC, Inc (Hamamatsu, Japan). Majority of the mature adult mice are ICR strain (ranging 3–9 months of age). The animal experiment (with Submission Number 996) was approved by the Animal Ethics Committee of Wakayama Medical University. We confirm that all methods were carried out in accordance with relevant guidelines and regulations. Three types of mixed general anesthesia (Medetomidine, Midazolam, Butorphanol) were administered into abdominal cavity trans-peritoneally.

### Fluorescence imaging

Imaging device was utilized (IVIS LUMINA XR, Caliper Life Sciences Inc. Hopkinton, MA; Fig. 1A) and the setting for ICG was adjusted below;

Exposure time: auto, Excitation filter: 700 nm, Emission filter: ICG (810–875 nm), Lamp level: high, the size of “Field of view”: 7.5 × 7.5 cm.

The general anesthesia was performed. An absorbable suture (6-0 PDS; polydioxanone suture, Echicon Inc. NJ) was placed into the glans for traction. The 2 cm vertical skin incision was placed into lower abdomen. After incision in the peritoneum, the distended bladder was displayed into abdominal cavity. ICG was dissolved with the final concentration of 0.25 mg/ml and injected into the bladder using 27-gauge needle.

### Antegrade urethrography

After the general anesthesia, antegrade urethrography was performed with the same system (Fig. 1A). The size of “Field of view” was adjusted with 5 × 5 cm. To take a spot X-ray image, exposure time was 10 s. A 6-0 PDS suture was placed into the glans for traction. The 2 cm vertical skin incision was placed into lower abdomen. After the incision, the distended bladder was displayed into abdominal cavity and it was punctured with 23-gauge butterfly needle and the tube of the needle was fixed to abdominal muscle and skin with 6-0 PDS. Penis was also fixed with tension by stay suture. The mouse was placed oblique position and contrast agent (iopamiron 375; iopamidol, Bayel AG, Osaka, Japan) was injected (Fig. 1B). The surgical wound was subsequently closed with two layers, fascia and skin.

### Murine hypospadias-like model

The general anesthesia was administered trans-peritoneally. A 7-0 PDS suture was placed into the glans for traction. Ophthalmic scissor was used in the incision of ventral urethra. 7-0 PDS was used in urethroplasty. The detailed description of procedures to generate the urethral abnormality mimicking the hypospadias (hereafter described as hypospadias-like surgery) is also described in Results.

### Histology and immunohistochemical staining

Specimens were fixed overnight in 4% paraformaldehyde/phosphate buffered saline, dehydrated in methanol, and embedded in paraffin. 6 µm thick sections were prepared for Hematoxylin–Eosin stain or immunohistochemistry. Rabbit monoclonal anti-E-Cadherin antibody (1:100, BD Biosciences, San Jose, CA) was used in this study. An Alexa 488-conjugated goat anti-rabbit IgG was utilized as a secondary antibody in fluorescent staining.

### Murine urethral stricture model

The general anesthesia was administered trans-peritoneally. A 7-0 PDS suture was placed into the glans for traction. The blind 7–0 PDS suture for urethra was performed so that the sutured 7-0 PDS was passed through 3 and 9 o’clock position. The detailed description of urethral stricture model is also described in Results.
